# Recent Advances In Silicon Carbide Chemical Mechanical Polishing Technologies

**DOI:** 10.3390/mi13101752

**Published:** 2022-10-16

**Authors:** Chi-Hsiang Hsieh, Che-Yuan Chang, Yi-Kai Hsiao, Chao-Chang A. Chen, Chang-Ching Tu, Hao-Chung Kuo

**Affiliations:** 1 Department of Photonics, Institute of Electro-Optical Engineering, National Yang Ming Chiao Tung University, Hsinchu 30010, Taiwan; 2 Institute of Pioneer Semiconductor Innovation, Industry Academia Innovation School, National Yang Ming Chiao Tung University, Hsinchu 30010, Taiwan; 3 Semiconductor Research Center, Hon Hai Research Institute, Taipei 11492, Taiwan; 4 Department of Mechanical Engineering, National Taiwan University of Science and Technology, Taipei 10607, Taiwan

**Keywords:** Silicon Carbide (SiC), chemical mechanical polishing (CMP), hybrid CMP, post-CMP cleaning

## Abstract

Chemical mechanical polishing (CMP) is a well-known technology that can produce surfaces with outstanding global planarization without subsurface damage. A good CMP process for Silicon Carbide (SiC) requires a balanced interaction between SiC surface oxidation and the oxide layer removal. The oxidants in the CMP slurry control the surface oxidation efficiency, while the polishing mechanical force comes from the abrasive particles in the CMP slurry and the pad asperity, which is attributed to the unique pad structure and diamond conditioning. To date, to obtain a high-quality as-CMP SiC wafer, the material removal rate (MRR) of SiC is only a few micrometers per hour, which leads to significantly high operation costs. In comparison, conventional Si CMP has the MRR of a few micrometers per minute. To increase the MRR, improving the oxidation efficiency of SiC is essential. The higher oxidation efficiency enables the higher mechanical forces, leading to a higher MRR with better surface quality. However, the disparity on the Si-face and C-face surfaces of 4H- or 6H-SiC wafers greatly increases the CMP design complexity. On the other hand, integrating hybrid energies into the CMP system has proven to be an effective approach to enhance oxidation efficiency. In this review paper, the SiC wafering steps and their purposes are discussed. A comparison among the three configurations of SiC CMP currently used in the industry is made. Moreover, recent advances in CMP and hybrid CMP technologies, such as Tribo-CMP, electro-CMP (ECMP), Fenton-ECMP, ultrasonic-ECMP, photocatalytic CMP (PCMP), sulfate-PCMP, gas-PCMP and Fenton-PCMP are reviewed, with emphasis on their oxidation behaviors and polishing performance. Finally, we raise the importance of post-CMP cleaning and make a summary of the various SiC CMP technologies discussed in this work.

## 1. Introduction

Silicon Carbide (SiC), a wide-bandgap semiconductor material, has attracted tremendous attention from both academics and industries in recent years. SiC possesses outstanding electrical properties for the power device application, including high breakdown electric field (2.5 × 10^6^ V cm^−1^), high thermal conductivity (5 W cm^−1^ K^−1^), high operating temperature (low intrinsic carrier concentration up to 900 °C) and high saturation drift velocity (2 × 10^7^ cm s^−1^) [1]. Although there have been more than 250 polytypes of SiC reported, the most commonly used polytypes are 4H-SiC, 6H-SiC and 3C-SiC. In particular, 6H-SiC and 4H-SiC wafers with diameters up to 8 inches have already been commercialized. Following our previous review of the SiC MOSFET fabrication process [1], in this review article, we focus on the recent advances in chemical mechanical polishing (CMP) technologies, which aim to provide atomically flat and subsurface damage-free SiC substrates for high-quality epitaxy and high-performance power device fabrication. Given that SiC is well-known for its exceptionally high hardness, brittleness and inertness, the current CMP of SiC usually suffers from high machining costs and low throughput. A comprehensive review of current CMP technologies may pave the way for further investigation to attain more efficient and more cost-effective SiC CMP.

Nowadays, a typical production process from the SiC boule to SiC substrates includes cropping/blocking, creating flat/notch, slicing, edge grinding, laser marking, lapping, grinding, CMP, post-CMP cleaning, inspecting and packaging (Table 1). The lapping and grinding steps involve mechanical polishing by using a diamond abrasive slurry or a diamond grinding wheel. The following CMP can effectively remove the machinery marks and subsurface damages induced by the lapping and grinding steps. Particularly, the effectiveness of surface oxidation plays a critical role in the SiC CMP. Therefore, the oxidation behavior on the Si-face and C-face surfaces of a SiC wafer and how it affects the CMP efficiency will be discussed. Finally, a summary of the emerging hybrid CMP technologies will be made and the importance of post-CMP cleaning will be pointed out.

## 2. Various SiC CMP Technologies

**Figure 1:**
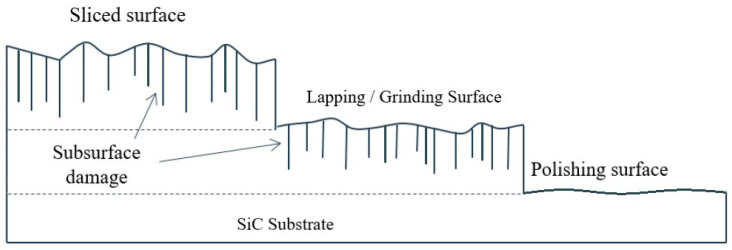
Illustrations of surface roughness and subsurface damage depth at the slicing, lapping, grinding and CMP steps of the SiC wafer production process [3].

Owing to the high hardness and high brittleness of SiC, diamond abrasives are commonly used in the lapping and grinding steps (Table 1). The resulting wafer surface topography, roughness and subsurface damage depth depend on the diamond abrasive size applied. In general, a smaller diamond abrasive size leads to a flatter surface, the smaller roughness and a shallower subsurface damage. The different levels of surface roughness and subsurface damage depth at different steps of the SiC wafer production process are illustrated in Figure 1. Compared to the lapping and grinding steps, since the hardness level decreases when the SiC surface gets oxidized, relatively soft and nanometer size abrasives, such as SiO_2_, Al_2_O_3_ and CeO_2_ nanobeads, are used during the CMP, resulting in angstrom scale flatness and subsurface damage free SiC surfaces. To date, for industrial applications, surface roughness (Ra) of Si-face < 0.1 nm and C-face < 0.4 nm are essential [2]. However, the relatively low-material-removal rate (MRR) remains the major issue of CMP. Compared to the MRR of Si CMP which is a few micrometers per minute, the current MRR of SiC CMP is only a few micrometers per hour. Therefore, how to optimize the process parameters and combine other energies into the system while maintaining acceptable surface quality has become the research focus of recent years. 

### 2.1. SiC CMP

**Figure 2:**
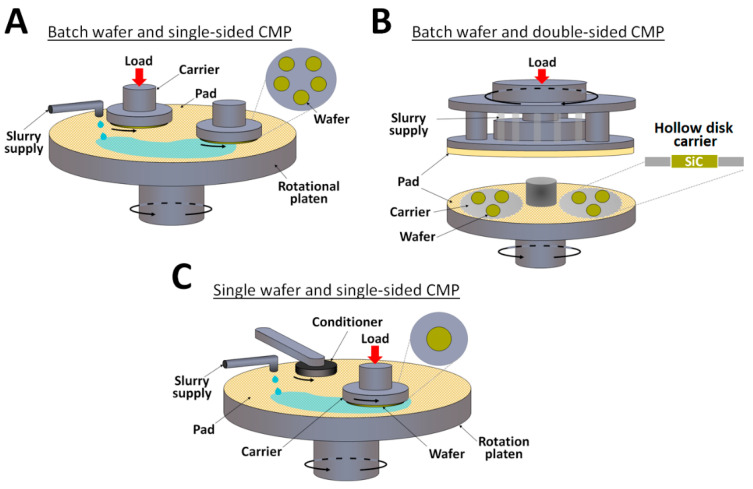
Different configurations of SiC CMP, including (**A**) batch wafer and single-sided CMP, (**B**) batch water and double-sided CMP and (**C**) single wafer and single-sided CMP. (Ref. CMC Materials Co.).

As the 100-mm, 150-mm and 200-mm SiC wafers are being commercialized for power device fabrication, the specifications of production-grade SiC wafers have become higher in order to meet the demand for more stable epitaxial growth. Three configurations of CMP have been developed to achieve higher MRR, lower surface roughness, less scratch and more uniform surface topography (Figure 2 and Table 2). For the batch wafer and single-sided CMP (Figure 2A), multiple wafers are loaded on the same polishing head by wax-mount or template-fix. For the batch wafer and double-sided CMP (Figure 2B), multiple wafers are loaded on the hollow disk carrier. Since there is no adhesive force between the wafer and carrier, the pressure and rotation speed are limited for preventing the wafers from slipping out, leading to relatively low MRR. For the single wafer and single-sided CMP (Figure 2C), the wafer tightly adheres to the polishing head by vacuum, allowing for higher pressure, higher rotation speed, and thus, higher MRR [2]. In practice, the double-sided CMP can serve as the first polishing step to polish the Si-face and C-face surfaces simultaneously. Since the C-face surface usually has a few times higher MRR than the Si-face surface, the single-sided CMP will continue to polish the Si-face surface until the production-grade level is achieved. Likewise, two single-sided CMP steps can be applied to, first, polish the Si-face and, then, the C-face for the graphene on C-face SiC application [4].

**Figure 3:**
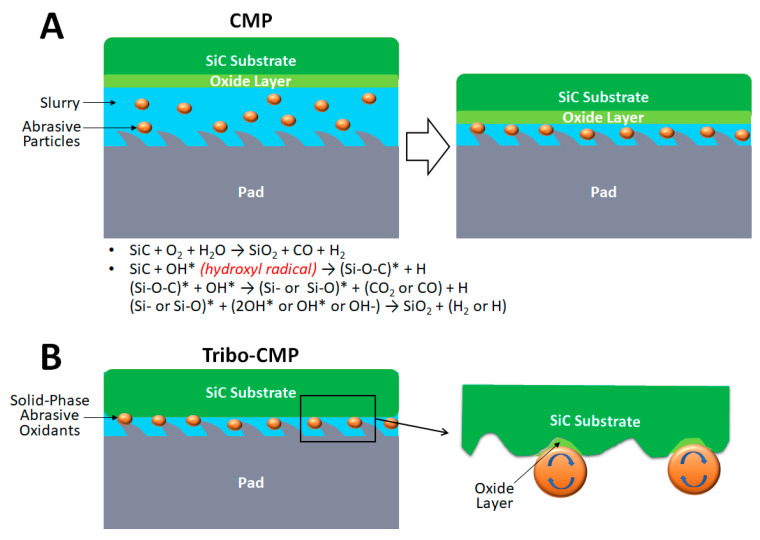
(**A**) Illustration of CMP mechanism. The oxidants in the slurry oxidize the SiC surface into a softer oxide layer which can be removed by the abrasive particles in the slurry and the conditioned CMP pad simultaneously. The chemical reactions of SiC oxidation, which are initiated by either oxygens or hydroxyl radicals, are shown [7,8]. Note that asterisk represents radicals. (**B**) Illustration of Tribo-CMP mechanism. In addition to the global oxidation taking place at the slurry and SiC interface, localized oxidation can also occur when the solid-phase abrasive oxidants roll on the SiC wafer surface.

Oxidants in the slurry provide the chemical driving force in the CMP system, as they produce a softer oxidized surface which can be removed by abrasives in the slurry and the conditioned CMP pad simultaneously (Figure 3A). To achieve a balance between surface oxidation and oxide layer removal, it is critical to understand the chemical and mechanical properties of the Si-face and C-face surfaces. In 2019, Lu et al. found that it is harder to remove C-face than Si-face, as revealed by the nano-indentation and nano-scratching methods [5]. The critical indentation load (Pc) of the Si-face (2.3 mN) and C-face (1.5 mN) surfaces and the effective indentation modulus (Er) of the Si-face (387.51 GPa) and C-face (266.02 GPa) surfaces were obtained by the Oliver-Pharr method. The nano-scratching test with a constant load of 4 mN at 1 µm s^−1^ scratch velocity was performed. The friction coefficients of the Si-face and C-face surfaces were measured to be 0.1778 and 0.2176, respectively. Most importantly, the resulting MRRs on the 6H-SiC Si-face and C-face surfaces were equal to 6.13 nm m^−1^ and 13.94 nm m^−1^, respectively, indicating that C-face is indeed easier to remove than Si-face [5]. In 2022, Shi et al. investigated the mechanical properties of different crystal orientations on a polished 4H-SiC wafer by using the nano-indentation and nano-scratching methods [6]. It was found that a larger elastic modulus leads to less material deformation, and therefore, a harder characteristic. At the penetration depth of 1200 nm, the Si-face surface showed elastic modulus and hardness equal to 305.5 GPa and 29.77 GPa, respectively. In contrast, the C-face surface showed elastic modulus and hardness equal to 476.7 GPa and 37.62 GPa, respectively, at the same penetration depth. Furthermore, the residue depth of indentation was 12.22 nm on the Si-face and 25.67 nm on the C-face, indicating more elastic recovery on the Si-face [6].

The SiC oxidation rate is strongly dependent on the crystal orientation. In 2011, Nitta et al. reported 4H-SiC MRR of 62 nm h^−1^ and 34 nm h^−1^ by using hydrogen peroxide (H_2_O_2_) and periodic acid (H_5_IO_6_) as the oxidants in a pH 10 slurry environment, respectively [7]. If using colloidal silica slurry alone, the MRR was only 12 nm h^−1^. Furthermore, the MRR increased from 47 nm h^−1^ to 106 nm h^−1^ after adding amine (piperazine) into the mixture of H_2_O_2_ and colloidal silica slurry. Without the amine component in the slurry, the oxide layer, which is formed through the Si radicals or Si−O−C radicals on the SiC surface, was mechanically removed by colloidal silica. In contrast, with the amine additive, it became the active oxide layer composed of Si-amine complexes to be mechanically removed by colloidal silica [7]. In 2013, Pan et al. proposed another chemical reaction path between H_2_O_2_ and SiC [8]. The 6H-SiC MRR increased from 80 nm h^−1^ to 105 nm h^−1^ with the H_2_O_2_ concentration increased from 3 wt% to 6 wt%. Adding alkali into the existing slurry (6 wt% H_2_O_2_ and 30 wt% colloidal silica) allows more hydroxyl ions into the system to enhance the chemical reaction. The CMP results showed 120% and 56% MRR increases with 0.5 wt% KOH and 0.5 wt% MEA added into the slurry, respectively [8]. In 2017, Chen et al. analyzed the chemical composition at the 6H-SiC surface by using X-ray photoelectron spectroscopy (XPS) under the conditions of as-received, pre-polished, after dipping in KMnO_4_ solution and after dipping in KMnO_4_ and KOH solutions sequentially with varied pH values [9]. The significant decrease in Si-O_x_-C_y_ and Si-O_2_ in the Si 2p spectra suggests native oxide removal from the as-received to pre-polish condition. Subsequently, the total oxide concentration increased dramatically after being dipped in KMnO_4_ solution and then decreased sharply after KOH treatment. The absence of the Si-O_2_ peak further confirms that the oxide layer can be dissolved in KOH [9].

In 2020, Tsai et al. proposed a mechanochemical CMP process (Tribo-CMP) [10]. In addition to the global oxidation taking place at the slurry and SiC interface, localized oxidation can also occur when the abrasive oxidants roll on the SiC wafer surface (Figure 3B). In this study, the water-dispersible abrasive oxidants composed of fullerene (C60) and β-cyclodextrin (β-CD) with particle sizes of 236 nm were used. During the CMP, the polishing pressure was 5.6 psi and the rotation speed was 90 rpm with a felt pad on the c-face. When using 0.01 wt% C60/β-CD abrasive oxidants in combination with 1 wt% 17-nm SiO_2_ particles, the resulting MRR was 49.9% higher than the control group, which only used 1 wt% 17-nm SiO_2_ particles. However, surface roughness (Sa) also increased around two fold for the test group, indicating the effect of non-uniform abrasion [10]. In 2021, Qi et al. investigated five solid-phase abrasive oxidants, including Na_2_CO_3_-1.5H_2_O_2_, KIO_3_, KClO_3_, KMnO_4_ and NaOH, for Tribo-CMP on 6H-SiC [11]. The SiC surface can be oxidized by oxygen generated from the decomposition of oxidants, along with friction-induced heat and water in the slurry formulation or ambient air. The solid-phase oxidants were sprayed on the polyurethane pad and the polishing experiments were conducted under the conditions of polishing pressure = 2 psi, polishing platen rotation speed = 60 rpm and polishing time = 90 min. A MRR of more than 1 µm h^−1^ can be consistently obtained for all five oxidants, but the resulting surface roughness was unsatisfactory. Meanwhile, obvious surface scratches and pits can be found under SEM inspection [11]. In addition to the solid-phase abrasive oxidants described above, in 2021, Ni et al. applied synthetic SiO_2_/CeO_2_ abrasives for CMP on the Si-face surface, under the conditions of polishing pressure = 3.5 psi and rotation speed = 90 rpm with a polyurethane pad [12]. The MRR equal to 0.451 µm h^−1^, 1.207 µm h^−1^ and 1.258 µm h^−1^ and the surface roughness (Ra) equal to 0.227 nm, 0.216 nm and 0.242 nm were obtained for the SiO_2_, SiO_2_/CeO_2_ and CeO_2_ abrasives, respectively. This result indicates that the synthetic SiO_2_/CeO_2_ abrasives with softer SiO_2_ cores (particle size: 80 nm to 90 nm) and harder CeO_2_ shells (shell thickness: 10 nm to 30 nm) not only generate a smaller surface roughness than pure CeO_2_ abrasives, but also provide a connection path to oxidized wafer surface through the Ce-O-Si bonds, which increases the sheer force during the CMP process [12].

The electrostatic interaction between the SiC wafer and abrasive particles is another critical factor to obtain an atomically flat surface with high MRR. Within a certain slurry pH range, the abrasive particles electrostatically adhere to the SiC wafer surface. Although the attached particles can provide a consistent mechanical force to remove the oxide layer, it might affect the oxidation efficiency if the particles remain at the same position and cannot be removed by the pad asperity. On the contrary, when the abrasive particles are electrostatically repelled away from the SiC wafer surface, the chance of having defects becomes higher and the MRR becomes lower, thus, requiring more concentrated slurry to achieve comparable polishing results. In 2007, Singh et al. determined that the iso-electric point (IEP) of SiC in deionized water is 5 [13]. Therefore, SiC carries a positive zeta-potential at a pH lower than the IEP. However, the IEP might shift to 3.6 when adding dispersant into deionized water, indicating that the IEP of SiC varies with the slurry composition. In 2015, Chen et al. investigated how the zeta-potential of potassium permanganate influences the performance of CMP based on SiO_2_ and CeO_2_ slurry particles [14]. Positive zeta-potentials were obtained for the pH range from 2 to 8 in the CeO_2_-based slurry, but there was no positive zeta-potential observed in the SiO_2_-based slurry for the pH range from 2 to 10. Under the conditions of polishing pressure = 4 psi, rotation speed = 90 rpm and potassium permanganate slurry pH = 2, the MRR and surface roughness (Ra) on the Si-face surface of 6H-SiC were measured to be 1.089 µm h^−1^ and 0.11 nm, respectively, when the polyurethane pad and CeO_2_ abrasives were used. Chen et al. further explained that abrasive particles with negative surface charges would easily attach to the SiC wafer surface. The attached abrasive particles then formed a blocking layer during the surface oxidation process, thus, lowering the MRR [14]. In 2021, Wang et al. demonstrated a high MRR of 1.4 µm h^−1^ with surface roughness (Ra) of 0.105 nm on the Si-face surface of 4H-SiC, by using an Al_2_O_3_ abrasive-based potassium permanganate slurry with pH = 2 [15]. The experiment was conducted under the conditions of polishing pressure = 6 psi and rotation speed = 90 rpm. However, the MRR continuously decreased to 1.1 µm h^−1^ when the pH increased to 12. The surface roughness (Ra) can be further improved from 0.105 nm to 0.066 nm after the second polishing step using the slurry composed of H_2_O_2_ oxidant, V_2_O_5_ catalyst and SiO_2_ abrasive [15]. It is worth noting that the electrostatic interaction plays a critical role not only at the wafer and abrasives interface, but also in the dispersion of abrasive particles. The optimization of abrasive particle size distribution in the slurry can effectively lower the surface scratches [16].

Compared to the commonly used liquid abrasive slurry, the fixed abrasive CMP technique provides higher mechanical force for higher MRR. In 2022, Wang et al. investigated non-aqueous slurries based on various types of organic solvents, such as methanol, ethanol, ethylene glycol and glycerol, with an abrasive pad fixed with self-sharpening and agglomerated diamonds (particle size: 3 µm to 5 µm) [17]. Under the conditions of polishing pressure = 0.7 psi and rotation speed = 50 rpm, the ethanol test group shows the highest MRR equal to 14.38 µm h^−1^. Meanwhile, the methanol test group shows the lowest surface roughness (Sa) equal to 12.22 nm, which however, is still worse than typical SiC performance [17]. In 2021, Zhou et al. applied molecular dynamics to simulate the surface morphology, subsurface damage and temperature distribution in a fixed abrasive CMP environment [18]. It was found that high-quality CMP requires uniform abrasive sizes and frequent interactions among neighboring abrasives [18]. On the other hand, a semi-fixed abrasive can be another technique to achieve higher MRR with lower surface roughness. In 2015, Lu et al. fabricated a semi-fixed abrasive sol-gel (SG) CMP pad, which can resolve the issue of uneven particle protrusion [19]. By using biopolymers as the matrix material, abrasives can be semi-fixed and self-adjusted to the same datum plan when the pad touches the wafer surface. As a result, the surface roughness (Ra) was greatly improved to 1.79 nm. In contrast, if using a fixed abrasive CMP pad, the surface roughness (Ra) was 24.61 nm [19]. Following a similar concept, in 2022, Luo at el. proposed that the semi-fixed SG CMP pad based on SiO_2_-modified diamonds, which are considered as soft–hard mixed abrasives, can further reduce the surface damage of the SiC substrate [20].

In addition to the experimental works, in 2022, Kayanuma et al. applied the density functional theory (DFT), unrestricted B3LYP method and 6-31G(d) basis set to analyze the reaction between OH radicals and O atoms within 10 surfaces of Si and C atoms on both Si-face and C-face surfaces [21]. The calculation results show that Si-O-Si bonds are formed on the Si-face and C-O-Si bonds are formed on the C-face, indicating that the Si-C bonds on the C-face can be easily dissociated by OH radicals to form a softer and easier machine surface [21]. In 2022, Morishita et al. analyzed the reactions involving water and H_2_O_2_ on the Si-face and C-face surfaces of 2H-SiC by using an ab initio molecular dynamics (AIMD) simulation [22]. The simulation results indicated that the C-face has a higher oxidation rate than the Si-face when either water or H_2_O_2_ was used because the Si-face is saturated with hydroxyl or hydrogen groups in a very short time. The higher oxidation rate leads to a higher MRR during the CMP process [22].

In summary, the three SiC CMP configurations currently used in the industry are introduced and the significance of process integration is highlighted. Furthermore, the mechanical properties and oxidation behaviors on the Si-face and C-face surfaces are compared. We also raise the importance of electrostatic interaction and how it affects CMP efficiency. As pointed out in the literature [23], integrating hybrid energies into the CMP system can be an effective approach to enhance oxidation efficiency as well as MRR. Therefore, in the following sections, we focus on recent advances in electro-CMP (ECMP) and photocatalyzed-CMP (PCMP) and their variants, such as Fenton-ECMP, ultrasonic-ECMP, sulfate-PCMP, gas-PCMP and Fenton-PCMP.

### 2.2. SiC Electro-CMP (ECMP)

**Figure 4:**
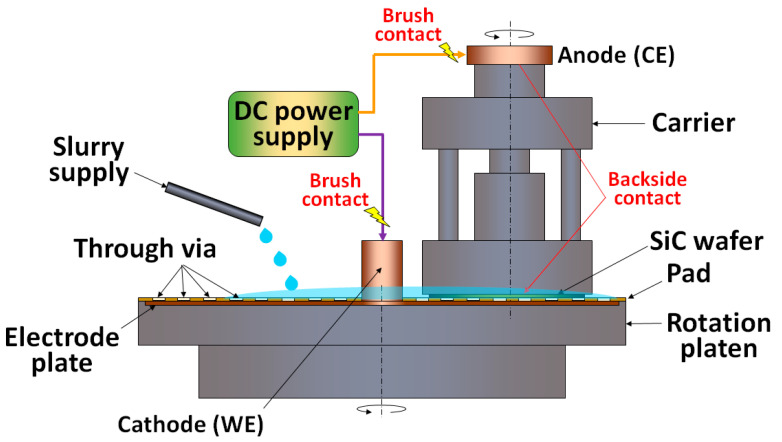
Typical setup for SiC ECMP.

The electro-CMP (ECMP) process has been widely investigated for the CMP of metal layers, such as Cu, W and Al, for many years [24]. For the SiC ECMP, the anodic oxidation can convert the hard SiC surface into a softer oxide layer, so that the abrasives composed of CeO_2_ or SiO_2_ can efficiently remove the oxide layer. In a typical ECMP setup (Figure 4), a working electrode (WE) is connected to a copper plate which has direct contact with the SiC wafer. A counter electrode (CE) is connected to the slip ring of the spindle and a reference electrode (RE) is connected to an Ag/AgCl plate. Since the ECMP relies on the wafer conductivity, N-type SiC wafers with low resistivity (~10^−3^ Ω·cm) are usually preferable to semi-insulating SiC wafers with high resistivity (~10^6^ Ω·cm). 

The potential dynamic polarization curve (PD curve) is often used to define the active region, passive region, transient region and trans-passive region, which can be used as references for setting the voltage range of ECMP. In 2019, Chen et al. analyzed the PD curves and found that there’s no clear passive region of 4H-SiC in different alkaline solutions [25]. On the other hand, the oxide layer thicknesses on both Si-face and C-face surfaces became thicker when higher voltages were applied to the 20 wt% NaNO_3_ solution. Furthermore, the C-face surface showed a significantly higher oxidation degree than the Si-face surface, and the difference increased with the larger voltage applied. By using nanoindentation tester, the hardness of Si-face decreased from 41.18 GPa to 2.53 GPa, and the hardness of C-face decreased from 32.1 GPa to 3.01 GPa after oxidation [25]. In 2015, Deng et al. compared the performance of diamond abrasive and CeO_2_ abrasive on an ECMP platform with 4H-SiC [26]. Under the conditions of polishing pressure = 3.74 kPa and rotation speed = 2000 rpm, a scratch-free surface was obtained by using CeO_2_ abrasive particles with a particle diameter of 190 nm. From the nanoindentation test, the surface hardness decreased from 34.5 GPa to 1.9 GPa after the anodic oxidation. The oxide layer growth rate was highly dependent on the applied voltage and the initial wafer surface roughness. For example, the oxide layer growth rates were equal to 0.6 µm h^−1^ and 0.92 µm h^−1^ when the applied voltages were equal to 5 V and 10 V, respectively. An oxide layer growth rate equal to 7.93 µm h^−1^ can be obtained on the diamond abrasive polished surface, which however, contained a subsurface damage layer [26].

**Figure 5:**
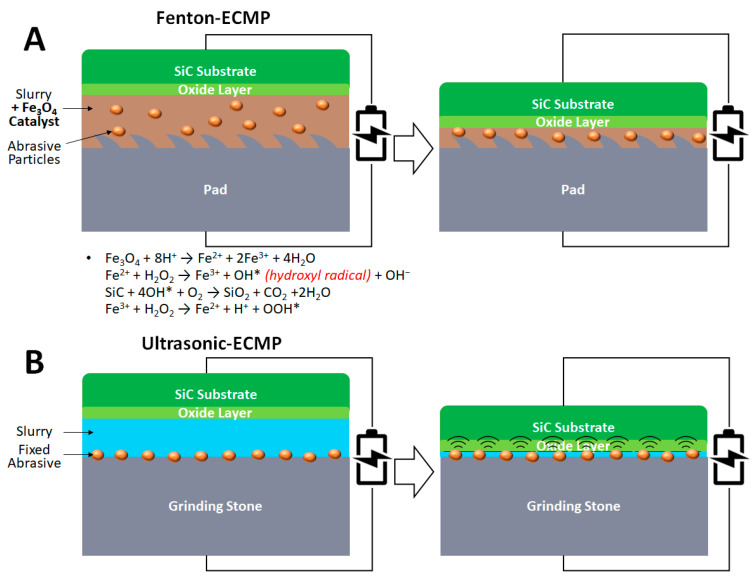
(**A**) Illustration of Fenton-ECMP mechanism. The oxidants and Fe_3_O_4_ catalyst in the slurry oxidize the SiC surface into a softer oxide layer which can be removed by the abrasive particles in the slurry and the conditioned CMP pad simultaneously. Under acidic conditions, Fe_3_O_4_ generates Fe^2+^ which undergoes a Fenton reaction with H_2_O_2_ to generate strong oxidant hydroxyl radical, denoted as OH* [27]. The oxide layer is removed by the abrasive particles in the slurry and the conditioned CMP pad simultaneously. (**B**) Illustration of ultrasonic-ECMP mechanism. After the anodic oxidation of SiC, the oxide layer is removed by a vibrating grinding stone with fixed abrasives.

In 2020, Deng et al. proposed that more hydroxyl radicals can be generated by the Fenton reaction, enhancing the oxidation of SiC CMP, during which ferrosoferric oxide (Fe_3_O_4_) is used as the catalyst [27]. The process is often called Fenton-ECMP (Figure 5A). In addition, in 2021, Deng et al. further studied the contribution of the Fenton reaction to the ECMP process [28]. Based on the oxidation activity on the C-face surface, the hydroxyl concentration increased by 196.24% and 135.03% when applying 3 V into the 2 wt% Fe_3_O_4_ and 5 wt% H_2_O_2_ slurry and 3 V into the 2 wt% Fe_3_O_4_ and 7.5 wt% H_2_O_2_ slurry, respectively. The hydroxyl concentration increased by 26.88% when the applied voltage was increased from 1.5 V to 3 V with the 2 wt% Fe_3_O_4_ and 5 wt% H_2_O_2_ slurry. Under the conditions of polish pressure = 0.04 MPa, rotation speed = 60 rpm, slurry flow rate = 30 mL min^−1^ and using a polyurethane pad, the MRR increased by 65.59% (from 238.36 nm h^−1^ to 394.7 nm h^−1^) and by 57.27% (from 281.93 nm h^−1^ to 443.4 nm h^−1^) after applying 1.5 V and 3 V into the 2 wt% Fe_3_O_4_ and 7.5 wt% H_2_O_2_ slurry, respectively. The initial wafer surface roughness (Ra) was 181.33 nm and then reduced to 31 nm to 36 nm by using the Fenton-ECMP. The lowest surface roughness (Ra) of 31.103 nm was achieved by applying 3 V into the 2 wt% Fe_3_O_4_ and 7.5 wt% H_2_O_2_ slurry [28].

In 2021, Yang et al. demonstrated a three-step ECMP process, which can potentially produce epitaxy-ready wafers directly from as-sliced wafers [29]. First, #8000 diamond fixed abrasive grinding stone (abrasive size: 1 µm) was used for ECMP at a current density of 20 mA cm^−2^ for 20 min to achieve MRR equal to 62 µm h^−1^, while decreasing the surface roughness (Sq) from 163.33 nm to 25.45 nm. Second, apply a #8000 CeO_2_ fixed abrasive grinding stone (abrasive size: 1 µm) at the current density of 10 mA cm^−2^ for 30 min to obtain 11 µm h^−1^ MRR and decrease surface roughness (Sq) to 0.82 nm. Finally, using the same #8000 CeO_2_ fixed abrasive grinding stone (abrasive size: 1 µm) at a passivation potential of 3 V for 1 h to achieve 6.3 µm h^−1^ MRR and decrease surface roughness (Sq) to 0.11 nm. A scratch-free and subsurface damage=free surface can be obtained, as confirmed by scanning white-light interferometry (SWLI) images and Raman images, respectively [29]. In 2022, Yang et al. proposed a slurry-less ultrasonic vibration-assisted ECMP (ultrasonic-ECMP) by placing a vibrator onto the lower polishing plate (Figure 5B) [30]. The working principle is based on the anodic oxidation of SiC, followed by the oxide layer removal by a fixed abrasive grinding stone. Under the conditions of polishing pressure = 30 kPa, wafer rotation speed = 50 rpm, platen rotation speed = 200 rpm, grinding stone oscillation rate = 2 mm s^−1^, ultrasonic vibration frequency = 35 kHz, ultrasonic vibration power = 500 W and voltage applied into the 1 wt% NaCl electrolyte = 25 V, the resulting MRR was equal to 14.54 µm h^−1^, which is significantly higher than the conventional CMP (0.05 µm h^−1^) and ECMP (3.2 µm h^−1^). Despite the high MRR, obvious surface roughness can be found on the wafer surface after the ultrasonic-ECMP process. For comparison, the wafer surface roughness (Sq) of ECMP and ultrasonic-ECMP treated wafers was 0.528 nm and 1.993 nm, respectively. The results raise the importance of vibration amplitude optimization in different electrolytes, which leads to different oxidation behaviors [30].

In summary, although the ECMP technologies have the limitation of being only suitable for N-type SiC wafers, compared to the conventional CMP described in the previous section, the ECMP technologies provide the potential to double-sided polishing which can increase the MRR due to higher incoming surface roughness from the mechanical polishing step. Furthermore, after the anodic oxidation, similar surface hardness levels on the Si-face and C-face surfaces may lead to similar MRRs, facilitating the CMP process integration in the future.

### 2.3. SiC Photocatalyzed-CMP (PCMP)

**Figure 6:**
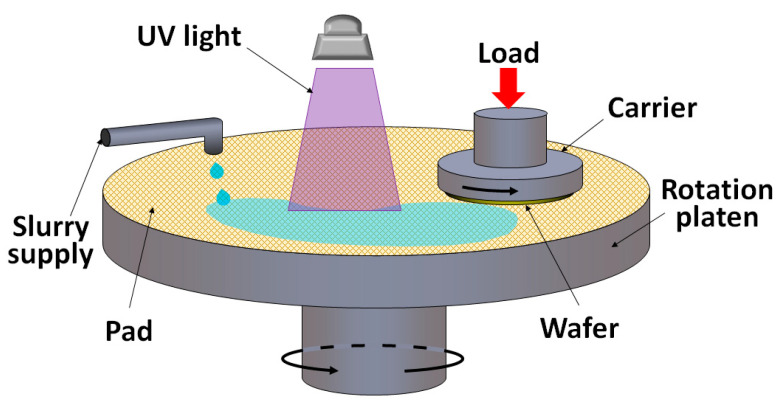
Typical setup for SiC PCMP.

**Figure 7:**
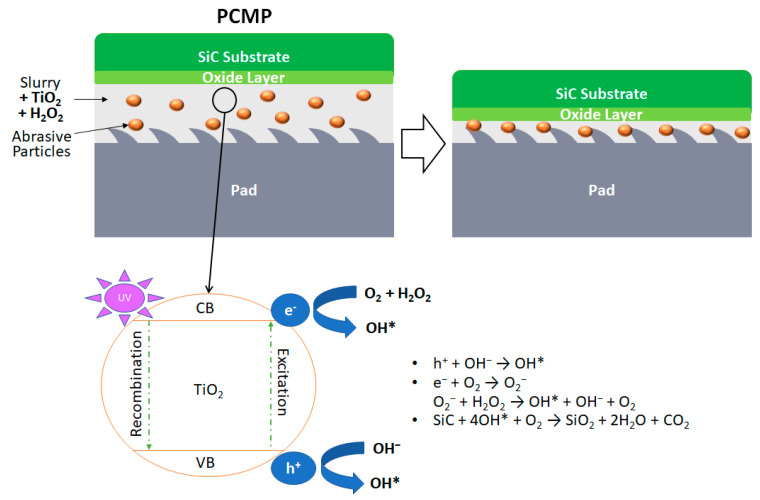
Illustration of PCMP mechanism. Upon irradiation of UV light, the photocatalytic TiO_2_ particle reacts with O_2_, H_2_O and OH^−^ in the slurry to generate strong oxidant hydroxyl radical, denoted as OH* [33]. The oxide layer is then removed by the abrasive particles in the slurry and the conditioned CMP pad simultaneously.

Photocatalyzed-CMP (PCMP) adds an additional oxidation source by applying UV light to highly photocatalytic particles, such as TiO_2_. The hydroxyl radicals generated by the photocatalytic process enhance the oxidation efficiency as well as the MRR (Figure 6 and Figure 7). In 2018, Yuan et al. proposed that photocatalysts, UV light, electron capturers and an acidic environment are the key factors in PCMP [31]. The MRR and surface roughness (Ra) equal to 0.96 µm h^−1^ and 1.95 nm, respectively, on the 4H-SiC surface, can be obtained by using synthetic fibrous polymer pads and applying UV light on the slurry consisting of TiO_2_ and H_2_O_2_ at pH = 2, polishing pressure = 0.025 MPa and rotation speed = 60 rpm. Compared to the control groups using TiO_2_ + H_2_O_2_ + pH 2 and TiO_2_ + UV + pH 2, the experimental group using TiO_2_ + UV + H_2_O_2_ + pH 2 yields the best polishing result [31]. In 2021, Wang et al. introduced a sulfate radical-based advanced oxidation process which applies sulfate (K_2_S_2_O_8_) into the PCMP system (sulfate-PCMP) [32]. Similar to the functionality of H_2_O_2_, the sulfate tends to generate SO_4_^−^ radicals during the PCMP process. Under the conditions of 3 wt% sulfate, 0.02 wt% TiO_2_ and pH 6 in the slurry, the highest MRR can be obtained [32].

**Figure 8:**
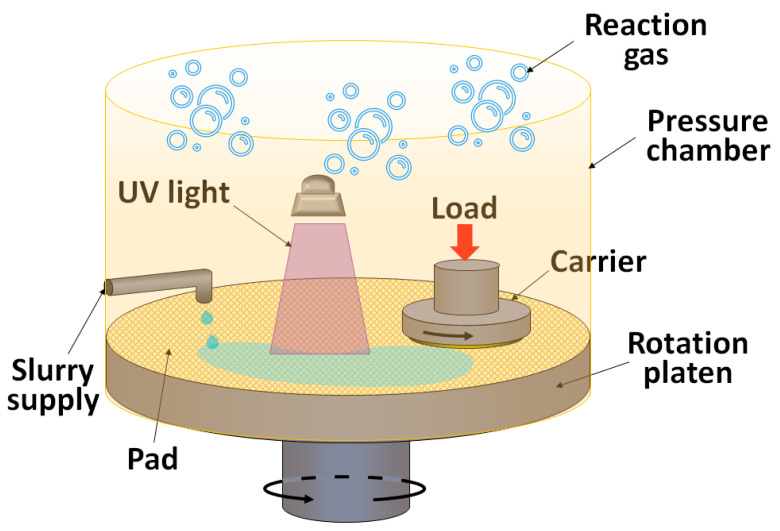
Typical setup for SiC gas-PCMP.

In 2021, Yin et al. developed a new PCMP method, in which the PCMP was conducted in an enclosed chamber filled with different compositions of gases, such as air, O_2_ and N_2_ (gas-PCMP, Figure 8) [34]. The highest MRR and dissolved oxygen (DO) amount were obtained on both Si-face surface (MRR = 43 nm h^−1^ and DO = 57 mg L^−1^) and C-face surface (MRR =108 nm h^−1^ and DO = 57 mg L^−1^) with the oxygen partial pressure equal to 300 kPa. By adding 0.3 wt% TiO_2_ particles and UV illumination into the system, the MRR can be increased by 30% and 10% on the Si-face and C-face surfaces, respectively. The MRR on the Si-face surface was about 2.2-fold higher than open air CMP [34]. In 2021, Lu et al. proposed a UV-photocatalyzed-Fenton combined mechanism for CMP, aiming at increasing the hydroxyl concentration as well as the MRR (Fenton-PCMP) [35]. Under the conditions of polishing pressure = 0.04 MPa, rotation speed = 60 rpm, slurry flow rate = 33 mL min^−1^ and using a polyurethane pad, the highest MRR of 387.2 nm h^−1^ on the C-face surface was obtained from the UV + TiO_2_ (4 g L^−1^) + Fenton group. The phenomenon that UV illumination could lead to better surface roughness was observed not only for the UV + TiO_2_ (4 g L^−1^) + H_2_O_2_ (5 wt%) group and UV + TiO_2_ (4 g L^−1^) + Fenton group, but also for the UV + H_2_O_2_ (5 wt%) group and UV + Fenton group. Moreover, the MRR holds a negative relationship with the surface roughness in the UV-illuminated groups [35].

## 3. Post-SiC CMP Cleaning

As described in Table 1, the main purpose of the SiC post-CMP cleaning is to remove organic, metallic and abrasive contaminations resulting from the CMP process, so as to achieve high-quality epitaxial growth in the following step [36]. Owing to the chemical inertness of SiC, traditional post-CMP cleaning methods, such as RCA and sulfuric peroxide mix (SPM), whose working principles are based on oxidation followed by etching, become problematic. In 2008, Madani et al. proposed an idea to remove the foreign bodies by directly reacting with them instead of micro-etching the substrate [37]. Hydrogen cyanide (HCN) solution was used in the study, which can provide cyanide ions to react with the metal contaminants. After the 4H-SiC wafer was immersed in the 0.08 M CuCl_2_ and 0.08 M NiCl_2_ solution for 10 min, the wafer was sequentially cleaned by the APM (29 wt% NH_4_OH:30 wt% H_2_O_2_:H_2_O = 1:1:5), HPM (36 wt% HCl:30 wt% H_2_O_2_:H_2_O = 1:1:5) and HCN solutions. Based on the total reflection X-ray fluorescence (TXRF) results, the Cu and Ni ions were completely removed by cleaning with 80 °C HPM followed by 25 °C HCN [37].

Although metal ions can be removed by HCN, how to remove other kinds of foreign bodies simultaneously raises the complexity of cleaning agent design, as well as the concerns for integrating into current manufacturing facilities. In 2022, Cahue et al. developed a contact cleaning method involving a poly-vinyl alcohol (PVA) brush, which transfers cleaning chemistry to the substrate and provides a mechanical force for efficient contamination removal [38]. After immersing the 4H-SiC wafer in a pH 4 slurry containing 0.005 wt% Al_2_O_3_ abrasive particles, the cleaning efficiencies with a brush only and after the addition of encapsulating chemicals were investigated. The particle count of the incoming dried wafer was around 10,000 ea and decreased to 6000 ea with a brush and deionized water. When both brush and encapsulating chemicals were applied, the particle count significantly decreased to 2000 ea. A similar trend was observed when the PVA brush was replaced with a megasonic mechanical source (30 sec and 1.5 W cm^−2^) [38].

## 4. Conclusions and Prospects

In this review article, we discuss the production steps from the SiC boule to epitaxy-ready substrates. Three configurations of SiC CMP currently used in the industry and their advantages/disadvantages are compared. The mechanisms that cause different surface properties as well as oxidation behaviors on the Si-face and C-face surfaces are discussed. Hybrid CMP technologies, such as ECMP, Fenton-ECMP, ultrasonic-ECMP, PCMP, sulfate-PCMP, gas-PCMP and Fenton-PCMP, give rise to the potential of increasing MRR while decreasing surface roughness. The operation conditions and performance parameters of various CMP technologies discussed in this article are summarized in Table 3. Lastly, the importance of post-CMP cleaning to the following epitaxial growth is pointed out. Cleaning with heated HPM and HCN solutions sequentially or applying a PVA brush has been proven to be able to remove metal ions and abrasive particles effectively. Upon the foundation of this review article, the research topics worth further investigation are suggested as follows. (1) How to utilize the unique structures of synthetic abrasive particles to enhance the CMP or hybrid CMP performance. (2) The trend of integrating multiple forms of energy into CMP has been obvious. However, how the interactions among these energy forms lead to the improvement of MRR and surface roughness is still not quite clear and needs further investigation.

## Figures and Tables

**Table 1:** SiC wafer production steps and the purposes. (Ref. CMC Materials Co., Kaohsiung City, Taiwan).

Production Step	Purpose
Cropping/Blocking	To cut off the boule’s head and tail, creating a flat datum plane. To shape the boule’s body into the desired diameter.
Creating Flat/Notch	To create alignment of the ingot.
Slicing	To cut the ingot into wafers with the desired thickness.
Edge Grinding	To create a shaped edge, for reducing edge chipping risk and increasing epitaxial film uniformity.
Laser Marking	To create an identification number of each wafer.
Lapping	To make the wafer thickness uniform. To lower the total thickness variation resulting from the slicing step.
Grinding	To lower the surface roughness level to nanometer scale. To minimize subsurface damage depth by using diamond grinding wheel.
CMP	To lower the surface roughness level to angstrom scale. To remove grinding marks, scratches and subsurface damages.
Post-CMP Cleaning	To remove abrasives and other organic and metallic contaminations from the wafer surface.
Inspecting	To ensure the wafer surface qualities, such as scratch, haze and metal levels, particle count and topology, meet the required specifications.
Packaging	To prepare the wafer ready for the subsequent epitaxial application.

**Table 2:** Comparison between different configurations of SiC CMP. (Ref. CMC Materials Co.).

Single/Batch Wafer	Single/Double Sided CMP	Advantages	Disadvantages
Batch	Single	Widely used in the mass production of 100-mm and 150-mm SiC wafers.	Total thickness variation increases with CMP time. Same defects tend to appear on other wafers within the same batch.
Batch	Double	Low total thickness variation	Lowest MRR Different removal rates on Si-face and C-face surfaces
Single	Single	Suitable for the mass production of 200-mm SiC wafers Flexible local flatness control by multi-pressure zone method Highest MRR Membrane assisted wafer holding saves loading and unloading times	Highest initial capital investment

**Table 3:** Operation conditions and performance parameters, namely MRR and surface roughness, of the various CMP and hybrid CMP technologies discussed in this article.

	Energy Type	MRR (µm h^−1^)	Surface Roughness (nm)	Note	Ref.
CMP	Chemical Mechanical	Si-face: 6.4 C-face: 22.5	Si-face: 0.1 (Ra) C-face: 0.4 (Ra)	4H-SiC Pressure (psi) × rotation speed (rpm)/100 = 9 Al_2_O_3_ abrasive Polyurethane pad	[2]
ECMP	Electrical Chemical Mechanical	Si-face: 0.42 C-face: 3.62	Si-face: 0.23 (RMS)	4H-SiC Applied voltage = 10 V Pressure (psi) × rotation speed (rpm)/100 = 10.8 CeO_2_ abrasive for Si-face Diamond abrasive for C-face PET pad	[26]
Fenton-ECMP	Electrical Chemical (Fenton) Mechanical	C-face: 0.443	C-face: 31.1 (Ra)	6H-SiC Applied voltage = 3 V Pressure (psi) × rotation speed (rpm)/100 = 3 SiO_2_ abrasive Polyurethane pad	[28]
Ultrasonic-ECMP	Electrical Chemical Mechanical (ultrasound)	14.54	1.993 (Sq)	4H-SiC Applied voltage = 25 V Vibrator: 500 W and 35 kHz Pressure (psi) × rotation speed (rpm)/100 = 8.7 Fixed abrasive pad	[30]
PCMP	Chemical Mechanical UV	0.96	1.95 (Ra)	4H-SiC Pressure (psi) × rotation speed (rpm)/100 = 3.6 Fibrous polymer pad	[31]
Gas-PCMP	Chemical Mechanical UV	Si-face: 0.07	Si-face: 0.98 (Ra)	4H-SiC O_2_ (300 kPa) + SiO_2_ abrasive + TiO_2_ photocatalyst Pressure (psi) × rotation speed (rpm)/100 = 2.4 Polyurethane pad	[34]
Fenton-PCMP	Chemical (Fenton) Mechanical UV	C-face: 0.387	C-face: 5.07 (Ra)	4H-SiC Pressure (psi) × rotation speed (rpm)/100 = 3.48 Polyurethane pad	[35]

## Data Availability

Not applicable.
